# Modeling Diffractive Lenses Recording in Environmentally Friendly Photopolymer

**DOI:** 10.3390/polym9070278

**Published:** 2017-07-12

**Authors:** Roberto Fernández, Víctor Navarro-Fuster, Francisco Javier Martínez, Sergi Gallego, Andrés Márquez, Inmaculada Pascual, Augusto Beléndez

**Affiliations:** 1 I.U. Física Aplicada a las Ciencias y las Tecnologías Universidad de Alicante, P.O. Box 99, E-03080 Alicante, Spain; roberto.fernandez@ua.es (R.F.); victor.navarro@ua.es (V.N.-F.); fj.martinez@ua.es (F.J.M.); andres.marquez@ua.es (A.M.); pascual@ua.es (I.P.); a.belendez@ua.es (A.B.); 2 Departmento de Física, Ing. de Sistemas y Teoría de la Señal, Universidad de Alicante, P.O. Box 99, E-03080 Alicante, Spain

**Keywords:** diffractive optics, optical recording materials, photopolymers, diffusion model, spatial light modulation

## Abstract

The improvements made in diffusion models simulating phase image recording in photopolymers enable the optimization of a wide range of complex diffractive optical elements (DOEs), while the miniaturization of spatial light modulators makes it possible to generate both symmetric and non-symmetric DOEs. In addition, there is increasing interest in the design of new friendly recording materials. In this respect, photopolymers are a promising material due to their optical properties. In this paper, we show a procedure to record diffractive spherical lenses using a nontoxic optimized photopolymer. To achieve this goal, we followed three steps: first, the chemical optimization for DOE recording; second, the recording material characterization to be simulated by a three-dimensional diffusion model; and third, the evaluation of the coverplating for the conservation of the DOE.

## 1. Introduction

Recently, a new hot topic known as “green photonics” appeared on the optics scene and is developing in leaps and bounds. Green photonics combines applications for generating and saving energy, reducing greenhouse gases, efficiently generating light, and the use of biodegradable materials. The associated technologies include photovoltaic, light emitting diode illumination, optical sensors, energy-efficient communication technologies, energy- and resource-efficient laser production processes, as well as non-contaminating materials [1,2]. In this sense, many efforts have been made to substitute the materials used in optics and photonics with less-contaminating ones. Polymers are one of the most interesting materials for recording diffractive and photonic elements [3,4]. In particular, photopolymers have become one of the photosensitive materials with the greatest ability to develop systems and photonic devices of considerable interest today [5].

Photopolymer recording materials are highly versatile in terms of their composition, reliability, and properties, making them one of the best recording media for holographic storage applications and an ideal candidate for the fabrication of photonic devices such as two-dimensional structures, waveguides, or diffractive optical elements [6,7,8]. However, one of the main drawbacks of these materials is the high toxicity of some of their components, so great efforts have been made to find alternative “greener” materials [9,10]. Additionally, the ability to modulate an incident wave using a spatial light modulator makes it possible to generate diffractive optical elements in many applications in photonics, communications, holographic storage, or information processing, as long as a recording material with suitable characteristics (e.g., photopolymers) is available.

One of the photopolymers widely used in optics is based on polyvinyl alcohol/acrylamide (PVA/AA), and interesting results were obtained when this material was used to produce blazed gratings [11] or spherical lenses [9]. Nevertheless, the high toxicity of the main monomer—acrylamide—makes it necessary to find biocompatible photopolymer formulations with similar optical properties. One of the best candidates is a photopolymer called “Biophotopol” [12]. However, the dye in this material is less sensitive for recording elements at 532 nm; therefore, we substituted it with yellowish eosin, which exhibits maximum absorption near this wavelength. As the refractive index modulation of this compound is slightly less than that of PVA/AA, we modified the technique used to form layers in order to increase the thickness of the film and achieve a phase depth of more than 2π. In this paper, we present a comprehensive study of the recording of spherical lenses of different focal lengths in different chemical compositions of this biocompatible photopolymer, with and without coverplating.

## 2. Materials and Methods

The Biophotopol photopolymer is composed of sodium salt 5′-riboflavin monophosphate (RF) dye and triethanolamine (TEA) as photoinitiator system that absorbs light, generating free radicals. This initiates the radical polymerization reaction of the sodium acrylate (NaAO) monomer solved in poly(vinyl alcohol) (PVA), which acts as binder. In this work, we use 35 mL of poly(vinyl alcohol) (*M*_w_ = 130,000, hydrolysis degree = 87.7%) as a binder in the preparation. The RF dye does not present good absorption at 532 nm [12], for which the main recording materials are optimized. Therefore, we have substituted it by yellowish eosin (YE) solved in water: 8 g/L (0.0116 M). This component is not as biocompatible as RF, but its toxicity is low and the quantity used in one sample is 6 mg. The need of balancing green materials and high performance led us to sacrifice some of the biocompatibility to obtain a recording material able to work with the desired wavelengths. We have analyzed three different chemical compositions (detailed in Table 1) for this described application.

Around 5.0 g of the resulting solution was deposited on a 6.5 × 6.5 cm^2^ glass plate using the force of gravity and left inside a dark incubator (Climacell 111) with controlled humidity and temperature (*H*_r_ = 60 ± 5% and *T* = 20 ± 1 °C, respectively). When part of the water had evaporated (drying time 16 h), the thickness of the “solid” film decreased to 140 ± 5 μm. At this time, the plate was then ready for exposure. The thickness of the solid film was measured using an ultrasonic pulse-echo gauge (PositTector 200, Clemco, Skanderborg, Denmark) after exposure. To prevent any effects due to changes on the surface, we applied an index matching and cover plating system like that described in [4] using paraffin as an index matching agent.

**Figure 1:**
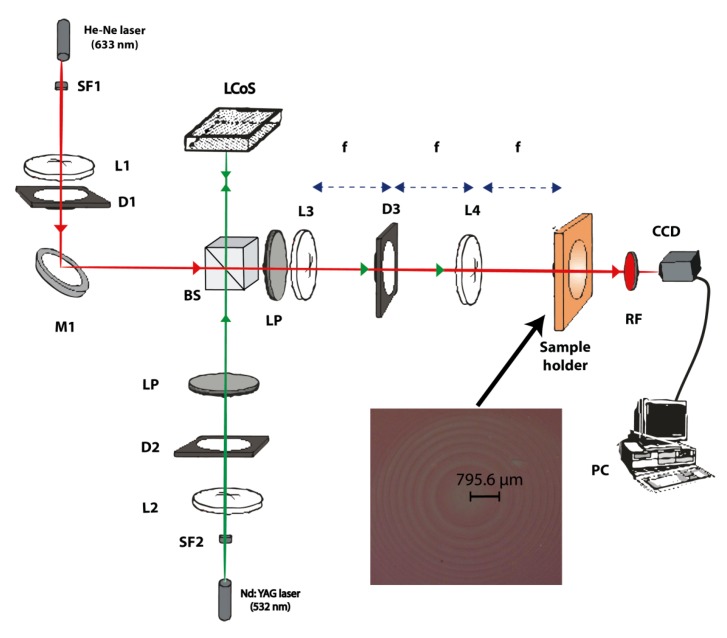
Experimental setup used to register and analyze in real-time the DOEs (diffractive optical elements, diffractive lenses) D: diaphragm; L: lens; BS: beam splitter; SF: spatial filter; LP: lineal polarizer; M: mirror; RF: red filter.

We used a hybrid digital-optical experimental setup, shown in Figure 1 [13]. The diffractive lenses were projected onto the material using a latest generation spatial light modulator based on a liquid crystal on silicon (LCoS, HOLOEYE Photonics AG, Berlin, Germany) microdisplay working in the amplitude mode. This setup was used to both record and analyze the diffractive optical lenses. A wavelength of 532 nm was used in the recording process, and the lenses were designed to be used at a wavelength of 633 nm. The system was designed to measure the performance of the lenses in real-time while the recording process was taking place. There were two arms in the setup: the recording arm, using a wavelength of 532 nm provided by a solid-state Verdi laser (Nd-YVO4, Coherent Inc., Santa Clara, CA, USA) at a recording intensity of 0.5 mW/cm^2^, and the analyzing arm, using a wavelength of 633 nm (at which the material does not absorb), provided by a He-Ne laser. Along the recording arm, the LCD was sandwiched between two polarizers (P), which were oriented at the appropriate angles (45° the first one and −45° the other) to produce amplitude-mostly modulation with a contrast of 20. A 4-F system was used to image the intensity transparency displayed on the reflective LCD onto the recording material. To generate the diffractive lenses, we used an LCoS-Pluto provided by Holoeye (LCoS, HOLOEYE Photonics AG, Berlin, Germany) with a resolution of 1920 × 1080 (HDTV) pixels and a pixel size of 8 × 8 μm^2^, previously characterized [14]. We projected lenses with two different focal lengths (13 and 60 cm). The analyzing arm was designed so that the beam of light incident onto the recording material is collimated. D1, a diaphragm, was used to limit the aperture of the collimated beam. A non-polarizing beam-splitter was included to make both beams of light (the recording and analyzing beams) follow the same path. A red filter was placed after the recording material so that the light incident on the final CCD camera came exclusively from the analyzing beam. The lens recorded on the photopolymer was responsible for focusing the 633 nm wavelength beam. The point spread function (PSF) generated by the diffractive lens was imaged onto the CCD camera. The magnification of our experimental setup using a 4-F system was controlled by the focal lengths of L3 and L4. In order to appreciate details in the PSF, a high dynamic range CCD camera was necessary. We used a pco.1600 model from pco.imaging (pco, Kelheim, Germany), a high dynamic 14 bits cooled CCD camera system with a resolution of 1600 × 1200 pixels and a pixel size of 7.4 × 7.4 μm^2^. The camera was also used on the plane of the recording material to evaluate the intensity pattern actually imaged from the LCoS plane.

The recording intensity distribution from the spatial light modulator in the amplitude regime is projected onto the material and produces the corresponding phase element. For the spherical lens with focal length f, we need to generate a convergent spherical wave front, where the phase depends on the quadratic value of the distance between the point and the lens centrum; thus, what we want to obtain at the first attempt can be written as:

(1) $$I(x,y)=\exp\left[j\frac{\pi }{\lambda \;f}\left(x^{2}+y^{2}\right)\right]$$

To obtain this phase distribution, we assume a linear response of the recording material converting an amplitude distribution generated by LCoS into a phase distribution. Therefore, Equation (1) must be wrapped to 2π value and normalized to the maximum intensity, *I*_0_. In this sense, we imaged an amplitude distribution with a maximum intensity in the middle, and there are a series of rings of zeros located at:

(2) $$r_{m}=\sqrt{m\left(2\lambda f\right)}$$

where *m* is a natural number, f is the focal length, and *λ* is the light wavelength. Moreover, we know that the maximum phase shift achieved for photopolymers in this spatial frequencies range is between 2π and 4π. Therefore, we have to wrap the phase; this desired intensity distribution is generated using an LCD.

**Figure 2:**
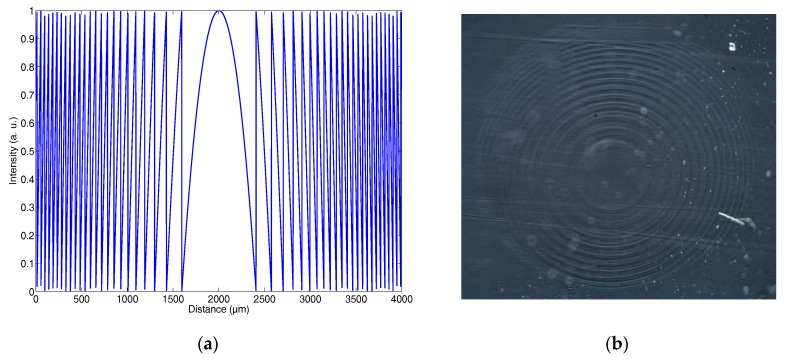
(**a**) Theoretical intensity distribution (horizontal cross section) sent to the liquid crystal on silicon (LCoS) for a focal length of 13 cm; (**b**) Photograph of the lens with a focal length of 60 cm recorded on material with composition 3.

Figure 2a shows a cross-section of the image sent to the LCoS for a lens with a focal length of 13 cm. It is important to point out that the focal length and the pixel size determine the maximum radius of the diffractive lens. For example, Figure 2a shows a 4-mm-diameter lens. In this Figure, just at the borders (the external part of the diffractive optical element, DOE), the distance between two consecutive maximums is less than 40 µm; in other words, five pixels to describe one ring. Furthermore, we know that there is crosstalk between two consecutive pixels, and this also affects the ring shape. Thus, with greater DOE diameters, at this focal length, it is impossible to reproduce the desired intensity distribution properly using our display due to the resolution limit. This result can produce an increase in the focal length of the recorded DOE. Figure 2b shows a diffractive lens with a focal length of 60 cm recorded on the photopolymer. For this lens, we used composition 3, and it was not coverplated in order to see the ring structure properly. For lenses with longer focal lengths, the profile of the recording intensity is more accurately defined and it is possible for the DOE to be larger.

## 3. Diffusion Model

In the case of spherical lenses, another dimension (related to the “*y*” variable) must be added to the standard diffusion models [8]. Then, the differential equations can be written as follows:

(3) $$\begin{array}{l} \frac{\partial M(x,y,z,t)}{\partial \;t}=\frac{\partial }{\partial \;z}D_{m}\left(t\right)\frac{\partial M(x,y,z,t)}{\partial z}+\frac{\partial }{\partial \;y}D_{m}\left(t\right)\frac{\partial M(x,y,z,t)}{\partial y}\\ +\frac{\partial }{\partial \;x}D_{m}\left(t\right)\frac{\partial M(x,y,z,t)}{\partial x}-F_{R}(x,z,t)M(x,z,t) \end{array}$$

(4) $$\frac{\partial P(x,y,z,t)}{\partial \;t}=F_{R}(x,y,z,t)M(x,y,z,t)$$

The polymerization rate depends on the reaction kinetics and the recording intensity; this dependence can be described by the following equation:

(5) $$F_{R}(x,y,z,t)=k_{R}(x,y,z,t)I{\left(x,y,z,t\right)}^{\gamma }=k_{R}(x,y,z,t)I{\left(x,y\right)}^{\gamma }e^{-\alpha (t)\gamma z}$$

where *I* is the recording intensity; *k* is the polymerization velocity; *γ* indicates the relationship between intensity and polymerization rate, 0.96; and *α* is the coefficient of light attenuation. The initial value of α(α(*t* = 0) = α_0_) can be obtained if the transmittance and the physical thickness of the layer are known.

The recording intensity distribution from the spatial light modulator in the amplitude regime is projected onto the material and produces the corresponding phase element. In the case of a spherical lens with focal length *f*, we need to generate a convergent spherical wave front, where the phase depends on the quadratic value of the distance between the point and the lens centrum, Equation (1)

Once we obtain the monomer and polymer concentrations, we can use the refractive index values to calculate the refractive index distribution during the recording process. The refractive index distribution can be measured using the Lorentz–Lorenz Equation as follows:

(6) $$\frac{n_{}^{2}-1}{n_{}^{2}+2}=\frac{n_{m}^{2}-1}{n_{m}^{2}+2}M+\frac{n_{p}^{2}-1}{n_{p}^{2}+2}P+\frac{n_{b}^{2}-1}{n_{b}^{2}+2}\left(1-M_{0}\right)$$

where *M*_0_ is the average initial value for the volume fraction of monomer, *n_p_* is the polymer refractive index, *n_m_* is the monomer refractive index, and *n_b_* is the binder refractive index. The last two parameters can be measured using a refractometer, and the value of *n_p_* can be obtained using the zero spatial frequency technique [15]. *M* takes the values of 0.15, 0.8, and 0.4 for compositions 1–3, respectively. The sample thicknesses are between 135 and 145 µm as measured by ultrasounds. The value of *k_R_* = 0.03 (cm^2^/mW)^−*γ*^ was measured by a technique based also on zero spatial recording [15], *γ* = 0.96 and the initial value of α (the attenuation due to the dye and described by Beer’s law) was 0.15 µm^−1^.

## 4. Results and Discussion

In this section, some important aspects concerning the formation of a DOE on Biophotopol using an LCoS as a master are discussed and analyzed. Taking into account previous results obtained recording sinusoidal low spatial frequency gratings and the zero spatial frequency limit with Biophotopol, we expected it to be more difficult to achieve a phase depth of 2π than it is with PVA/AA materials. First, we describe the recording of diffractive lenses of different focal lengths in order to check the validity of the model simulations. Then, different material compositions are studied with a view to achieving optimum optical behavior, and finally the effect of coverplating and using paraffin as an index matching agent on the formation and conservation of the diffractive lenses is briefly discussed.

### 4.1. Model Validation

**Figure 3:**
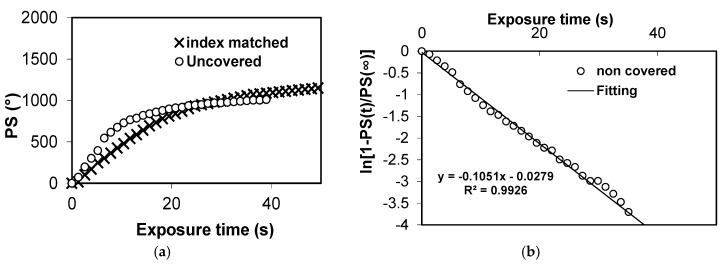
Phase shift (PS) at zero spatial frequency recording as a function of exposure time.

As mentioned in the introduction, we previously measured all the parameters introduced in the diffusion model by means of metrology experiments based on interferometric and diffractive techniques [15]. One of the key parameters is the polymerization rate, which is measured by zero spatial frequency experiments. In Figure 3, we present the experimental results obtained for chemical composition 1—the standard composition optimized for holographic application [12]. Figure 3a shows the variation in phase shift proportional to the movement of the interference fringes on the CCD camera [15]. In this Figure, we compared the results in the same layer with and without index matching. In both cases, the behavior is very similar. Figure 3b shows the fitting considering the Trommsdorff effect, whereby the polymerization rate decreases as the polymer concentration and viscosity of the material increase. As can be seen, the experimental results are very close to the theoretical predictions, and we obtain the initial value of the polymerization rate for this composition.

**Figure 4:**
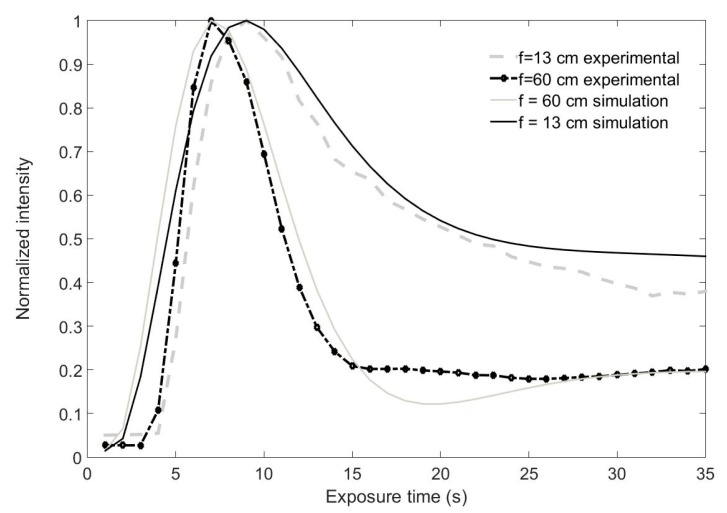
Normalized intensity at the focal point for two different focal lengths of 13 cm and 60 cm. Experimental data and simulations are represented.

Once we accurately determined all the parameters involved in DOE formation in the material, we recorded some spherical lenses. Due to the small size of the LCoS pixel, we could attempt to store shorter focal length lenses. In this case, we recorded lenses with a focal length of 13 and 60 cm. To be more realistic in our simulations, we introduced in the model the real intensity distribution captured by the CCD camera placed in the material plane. Both experimental data and the corresponding simulations provided by the model are presented in Figure 4. As can be seen, the agreement of the model with the experiments is very high. Both focal lengths present very similar behavior, as was also predicted for PVA/AA materials in [8]. The shorter focal length lens exhibits a slightly faster focalization, perhaps due to the greater influence of monomer diffusion. In this study, the spatial frequency of the gratings was higher than that found in other materials [8]. It is worth noting that in both cases, the phase depth achieved was greater than 2π, and in the case of a focal length of 13 cm, the final focal length was close to 4π. This interesting fact makes the material very appealing for additional applications [15,16].

### 4.2. Different Monomer Concentrations

**Figure 5:**
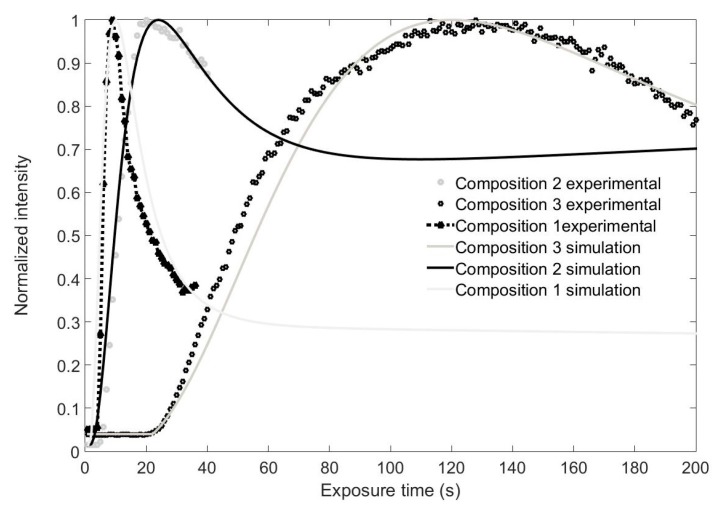
Normalized intensity as a function of recording time at the focal point for different material compositions: experiments and simulations. Focal length of 13 cm.

**Figure 6:**
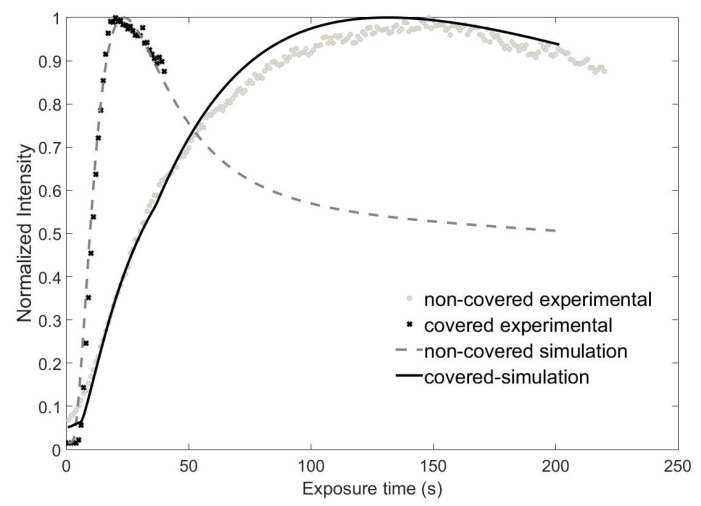
Normalized intensity as a function of recording time at the focal point for the same sample without cover-plate and covered and index matched: experiments and simulations. Focal length of 13 cm.

Chemical composition 1 exhibits very appealing properties for holographic and DOE applications. However, it is worth noting that due to its high monomer concentration, it is not very stable outside of the controlled incubator chamber. In other words, after a few hours, part of the monomer begins to precipitate, thereby affecting the transparency of the layer and increasing the scattering of light. This means that the time available in which to record holograms or DOEs is considerably reduced. Since it can be seen in Figure 5 that the phase depth is clearly larger than 2π with this thickness, we decided to reduce the monomer concentration. The results are presented in Figure 6 for a focal length of 60 cm. It can be seen that the recording time needed to achieve the maximum focalization power increases when the monomer concentration is reduced. Additionally, in the layers with the lowest monomer concentration, the inhibition period described and modeled by [17] may be clearly seen. In our diffusion model, this effect is not included, so we start to apply the model when the intensity starts to grow. It is important to point out that a phase depth of 2π is achieved in all the layers.

### 4.3. DOE Conservation

**Figure 7:**
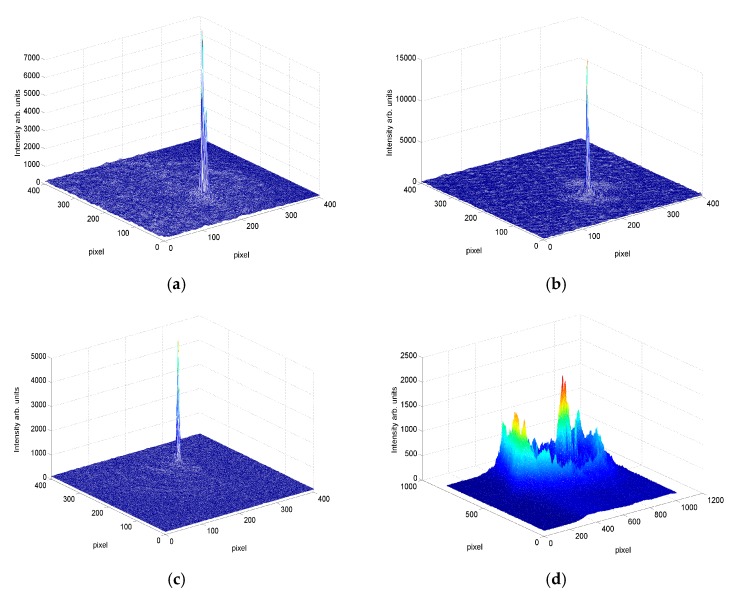
Point spread function (**a**) Just after recording for coverplated samples; (**b**) Just after recording for uncovered samples; (**c**) After 8 weeks for coverplated samples; (**d**) After 4 weeks for uncovered samples.

In order to improve the DOE conservation, we applied a cover-plate together with an index matching liquid. As we commented in relation to Figure 3, this sample architecture does not seem to affect the polymerization process, whereas it prevents the undesired effects of changes in the material surface thanks to the paraffin used as index matching agent. We compared the recording process of the lenses in both cases and their conservation over several weeks in dark conditions. During this time, the samples could be illuminated with red light because the material does not absorb at this wavelength. The formation of the lenses can be described analyzing Figure 7. This figure shows the intensity in the focal plane as a function of time during the recording of a diffractive lens with a focal length of 13 cm in composition 2. It can be seen that a maximum phase depth of 2π was reached faster with index matching. Additionally, the focalization power—the difference between maximum and initial intensity—was also greater with coverplating. 

In order to obtain a deeper insight, we analyzed the point spread function for both sample systems immediately after recording and one month later. Figure 7 shows the three-dimensional representation of the PSF for 140 µm-thick lenses with a focal length of 13 cm. Figure 7a,b correspond to the moment immediately after recording for coverplated and non-coverplated samples, respectively. Note that the pixel size of the CCD camera was 7.4 µm. Analyzing the results just after recording, it is clear that a high-quality DOE can be obtained using both types of samples. Figure 7c,d corresponds to the PSF one month later. As can be seen, coverplating provides a good conservation of the stored DOE over time, whereas in the case of uncovered samples, almost all the information disappears after four weeks. The composition used was C2, but we detected the same behavior with the other compositions. In both cases, we observed good focalization after recording; nevertheless, it is important to note that a secondary peak can sometimes appear when the index matching is not perfect, because some air remains between the cover and the photopolymer. Another important point to consider in order to reproduce the above experiments is that higher intensities are obtained at the focal point with diffractive lenses of shorter focal length due to the smaller area of the PSF; therefore, we reduced the intensity of the read out laser (633 nm in our case). In the case of Figure 7d, the focalization power was so weak that we increased the read out intensity, and so the differences between the background light and the focal point were not as high as in the other figures.

## 5. Conclusions

We have demonstrated a detailed method for recording and modeling the recording of spherical diffractive lenses onto a photopolymer with good focalization power and PSF using a hybrid optical-digital experimental set up with an LCoS display as a master. We improved their conservation and energetic sensitivity using a cover-plating and index matching system. The material exhibited good properties for focal lengths longer than 13 cm. Chemical compositions 2 and 3 were more stable, more reproducible, and could store DOEs for longer. On the other hand, their energetic sensitivity was lower and the phase depth did not reach the value of 4π required for some special applications like achromatic lenses. 

## Figures and Tables

**Table 1:** The three different chemical solutions of Biophotopol which were analyzed. NaAO: sodium acrylate; PVA: poly(vinyl alcohol); TEA: triethanolamine; YE: yellowish eosin.

Component	Composition 1	Composition 2	Composition 3
NaAO 81% (mL)	3.5	2	1
TEA (mL)	0.35	0.2	0.1
PVA F18-88 15% (mL)	35	35	35
YE (0.8% *w*/*v*) (mL)	0.75	0.75	0.75
